# Detecting change in a caregiver-mediated autism intervention using the Joint Engagement Rating Inventory

**DOI:** 10.4102/sajcd.v72i1.1102

**Published:** 2025-09-26

**Authors:** Zahra Dawood, Katlego Sebolai, Minkateko Ndlovu, Marisa Viljoen, Noleen Seris, Nokuthula Shabalala, Petrus J. de Vries, Lauren Franz, Michal Harty

**Affiliations:** 1 Division of Communication Sciences and Disorders, Department of Health and Rehabilitation Sciences, University of Cape Town, Cape Town, South Africa; 2 Center for Autism Research in Africa, Division of Child and Adolescent Psychiatry, Department of Psychiatry and Mental Health, University of Cape Town, Cape Town, South Africa; 3 Division of Child and Family Mental Health and Community Psychiatry, Department of Psychiatry and Behavioral Sciences, Duke University, Durham, United States

**Keywords:** Joint Engagement Rating Inventory (JERI), naturalistic developmental behavioural interventions (NDBI), caregiver coaching, signals of change, intervention response

## Abstract

**Background:**

The Joint Engagement Rating Inventory (JERI) can be used to measure response to early autism intervention. However, little is known about the utility of the JERI outside the United States, where it was developed. A South African study found the JERI to be a reliable and accurate measure of joint engagement and communication between young autistic children and their caregivers. The next step was to determine if the JERI could be used to detect changes in the behaviours of child and caregiver in response to intervention.

**Objectives:**

This proof-of-principle study aimed to evaluate whether the JERI could detect signals of change in the behaviours of child and caregiver in response to 12, 1-h naturalistic developmental behavioural intervention-informed caregiver coaching sessions.

**Method:**

A single-arm pre–post design was utilised. Standardised video-recorded caregiver–child interactions were completed before and after intervention. Two raters, blinded to intervention time-point, coded the JERI. Ten dyads completed coaching and video-recorded assessments. Data analysis included inter-rater reliability, Wilcoxon signed-rank test for paired samples and visual summaries.

**Results:**

Weighted Kappa values for 13 of the 16 JERI items indicated moderate to strong inter-rater agreement. Significant changes in symbol-infused joint engagement (*z* = −2.46, *p* = 0.01) and expressive language (*z* = −2.156, *p* = 0.03) were detected. Visual summaries showed change signals in 15 JERI ratings.

**Conclusion:**

Findings suggest that the JERI has the potential to detect change in the context of a caregiver-mediated intervention.

**Contribution:**

The JERI was shown here, for the first time in an African context, to be a potential outcome measure for early autism intervention research.

## Introduction

A lack of access to services and support for young autistic children is a growing public health concern globally (Lord et al., 2022). In particular, low- and middle-income countries (LMICs) are affected by limited availability of services (De Vries, 2016). South Africa is an upper-middle-income country significantly impacted by health disparities and income inequality as exemplified by a Gini coefficient of 0.67, indicating severe inequality (World Bank Group, 2025). These disparities directly impact the availability of and access to services and supports for young autistic children and their families (Mayosi & Benatar, 2014; Sulla, 2022). Work is underway to address the need for contextually appropriate autism early interventions in South Africa (Rieder et al., 2023; Schlebusch et al., 2020, 2024; Viljoen et al., 2024). For example, various evidence-based supports and interventions, including Parent Education and Training (PET) programmes and Naturalistic Developmental Behavioural Interventions (NDBI), are currently being studied by interdisciplinary teams at the Centre for Autism Research in Africa, based at the University of Cape Town (Schlebusch et al., 2020). Naturalistic Developmental Behavioural Interventions are a class of empirically based early intervention methods, derived from the principles of behavioural and developmental science (Schreibman et al., 2015).

Acknowledging the limited specialist workforce in South Africa, supports and interventions under development utilise task-sharing, which involves redistributing roles typically performed by specialist providers to non-specialists, including laypeople (Orkin et al., 2021). In caregiver-delivered interventions, task-sharing also allows for redistribution of roles to the caregivers themselves. A recent pilot study, conducted in collaboration with the Western Cape Department of Education, explored whether a NDBI caregiver-mediated intervention implemented by Early Childhood Development (ECD) practitioners, a non-specialist workforce employed by the Education Department, could be delivered with fidelity and whether signals of change in child and caregiver outcomes could be detected (Rieder et al., 2023). In the intervention, caregivers received 12, 1-h coaching sessions, and results supported the potential of task-sharing approaches in low-resource contexts such as South Africa to help address the early autism intervention gap (Rieder et al., 2023). A randomised effectiveness-implementation trial of this NDBI caregiver coaching approach is currently underway as a next step from the early work (Franz et al., 2024).

To measure child and caregiver response to NDBI caregiver-mediated intervention, behavioural coding approaches, including the Joint Engagement Rating Inventory (JERI), can be used (Adamson et al., 2019; Bertamini et al., 2021). The JERI contains 18 items on a 7-point Likert scale that characterise joint engagement, communication dynamics and shared topics between a young child and their caregiver (Adamson et al., 2019). The JERI is coded by trained raters who are blinded to assessment time-point (pre- versus post-intervention). While the JERI has almost exclusively been used in research conducted in high-income countries (HICs) (Mirenda et al., 2022; Settanni et al., 2024), in recent South African studies, the JERI has been found to be a reliable and accurate tool in a lab-based setting to quantify interactions between caregivers and their young autistic children (Ndlovu, 2022). Furthermore, smartphone recordings of caregiver–child interactions (CCI) were found to be technically feasible to code using the JERI (Ndlovu, 2024).

To expand on the caregiver-report and clinician-rated measurement approach used in the South African pilot by Rieder et al. (2023), this proof-of-principle study examined the JERI as a pre–post measure to quantify behavioural signals of change in child–caregiver dyads. Specifically, this study aimed to evaluate whether the JERI could detect signals of change in child behaviours, caregiver behaviours and dyadic engagement in young autistic children and their caregivers, after 12, 1-h NDBI-informed caregiver coaching sessions implemented by non-specialists.

## Research methods and design

### Study design and setting

A single-arm pre–post design was used for this proof-of-principle pilot study to assess signals of change in autistic children and their caregivers after 12, 1-h NDBI-informed caregiver coaching sessions implemented by ECD practitioners. The study was a collaboration between the Centre for Autism Research in Africa at the University of Cape Town and the Duke Centre for Autism and Brain Development at Duke University. Caregiver–child dyads were recruited from Western Cape Provincial Department of Education waiting lists for autism special education services. In South Africa, public special education schools are the third level of school placement in the education system, intended for learners with high-support needs (Nthibeli et al., 2022).

### Participants

As outlined in the pilot study by Rieder et al. (2023), inclusion criteria for autistic children and their caregivers were as follows: (1) the child was between 18 and 72 months of age; (2) the child’s family primary language was isiXhosa, isiZulu, Afrikaans or English; (3) the participant’s self-declared race was black person, coloured person or Indian person; (4) the child lived within an area served by the recruitment sites and was on the Provincial Department of Education waiting list; (5) the child met the DSM-5 criteria for autism spectrum disorder informed by the Autism Diagnostic Observation Schedule Second Edition (ADOS-2) administered by research-reliable clinicians (the child entered the study with diagnosis of autism, which was confirmed in the study); and (6) the caregiver was ≥ 18 years. Exclusion criteria for caregiver–child dyads were as follows: the child had (1) significant sensory or motor impairments, major physical abnormalities, the presence of a neurological disorder of known aetiology (e.g. Fragile X syndrome), history of serious head injury and/or neurological disease, or (2) the caregiver–child dyads were unable to attend assessments and the 12 coaching sessions. All families lived within a metropolitan area.

### Procedures

**FIGURE 1:**
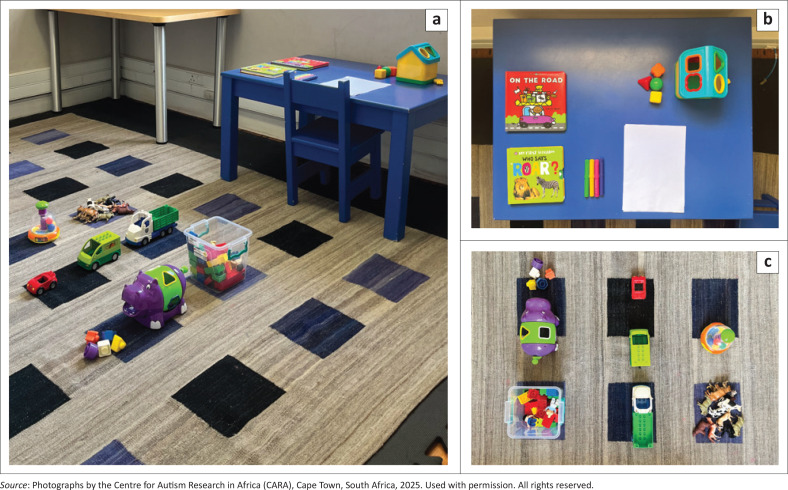
Low-cost play materials set-up for the caregiver–child interaction (CCI) protocol in a lab-based setting: (a) Room view, (b) table view and (c) floor view of play-material set-up.

After consenting to participate, the dyads were video-recorded during their baseline assessment, using a standardised CCI protocol. The CCI consisted of two consecutive 6-min free play sessions referred to as ‘Part one’ and ‘Part two’. In Part one, the caregiver was instructed to remain seated, while their child interacted with play materials in the room. In Part two, the caregiver was instructed to interact with their child in a way they would at home. After the caregiver–child dyads had completed 12 coaching sessions, the dyads attended a follow-up assessment. During the follow-up assessment, the standardised CCI protocol was repeated. The video recordings of the CCI were uploaded to Duke Box (a Health Insurance Portability and Accountability Act-compliant transfer and storage platform hosted by Duke University). All CCI occurred in the same room (see Figure 1 for material set-up for the CCI protocol).

### Measures

Sociodemographic characteristics of the child (age, self-declared race by caregiver, gender and home language) and caregiver (age, relationship to child, level of education, employment status and household income) were assessed. Autism-related behaviours were assessed using the ADOS-2, a semi-structured play-based assessment administered by a research-reliable clinician (Lord et al., 2012).

The JERI is a behavioural coding system used to characterise joint engagement, communication dynamics and shared topics between young children and their caregivers (Adamson et al., 2004, 2019). Sixteen JERI items were included in this study that capture descriptions of child joint engagement and social communication, caregiver’s ability to support and facilitate child activity and dyadic interaction (see Table 1). Joint Engagement Rating Inventory items are coded on a 7-point Likert scale with descriptive anchors at ratings of 1, 4 and 7 from videos of CCI (see Table 1). Data were coded by two research-reliable JERI raters who were blinded to whether CCI recordings were pre- or post-intervention. As per coding convention, a master rater was randomly assigned CCI videos for double coding to determine inter-rater reliability with the research-reliable JERI raters.

**TABLE 1:** Joint Engagement Rating Inventory items and their anchors.

Items	Anchors		
	1	4	7
1. Child unengaged	Almost always engaged with objects, people and/or symbols	Unengaged for approximately a third of the scene	Almost always unengaged
2. Object engagement	No episodes of engagement	Spends about a third of the scene in object engagement that is of moderate quality, or more briefly in a highly striking manner	Frequently in rich and varied episodes of object engagement
3. Child joint engagement	No episodes of joint engagement	Spends about a third of the scene in joint engagement that is of moderate quality, or more briefly in a highly striking manner	Frequently in rich and varied episodes of joint engagement
4. Supported joint engagement	No episodes of supported joint engagement state	Spends about a third of the scene in supported joint engagement that is of moderate quality, or briefly in supported joint engagement in a strikingly high-quality manner	Frequently in rich and varied episodes of supported joint engagement
5. Coordinated joint engagement	No episodes of the coordinated joint engagement state	Spends about a third of the scene in coordinated joint engagement that is of moderate quality, or briefly in coordinated joint engagement in a strikingly high-quality manner	Frequently in rich and varied episodes of coordinated joint engagement
6. Symbol-infused joint engagement	No episodes of the symbol-infused joint engagement state	Spends about a third of the scene in symbol-infused joint engagement that is of moderate quality, briefly in symbol-infused joint engagement in a strikingly high-quality manner	Frequently in rich and varied episodes of symbol-infused joint engagement
7. Child’s responsiveness to partner’s communication	Almost always resists or ignores bids	Responds to bids regularly, but not continually	Complies with and anticipates almost every bid
8. Child’s expressive language level and use	No expressive language	Produces many different single words during a scene with few or no word combinations	Fluent and frequent use of sentences
9. Child’s attention to caregiver	Does not pay attention to caregiver	Intermittently pays attention to caregiver	Frequently in rich and varied episodes of attending to the caregiver
10. Caregiver’s scaffolding	Provides minimal support for the child’s communication and/or actions on objects	Provides moderate levels of support	Continually supports and extends the child’s actions
11. Caregiver’s following in on child’s focus	Rarely follows in on the child’s current focus	Builds on the child’s focus on a regular, but not continual basis	Almost continually joins and acts to sustain the child’s interest
12. Caregiver’s affect	Tense, disruptive or affectively flat, to the point of being expressionless and subsequently very hard to read	Mellow or content as opposed to flat; affect does not impede communication, but neither does it enhance it	Smoothly modulated, appropriate and serves to enhance other modes of communication
13. Caregiver’s language facilitation	Rare use of language facilitation strategies	Language scaffolding strategies are employed in one third of the scene; or less time but of high quality; or more time of low quality	Continual use of a variety of language facilitation strategies
14. Caregiver’s communicative temptations	Never makes any communicative temptations	Communicative temptation strategies in one-third of the scene; or less time but of high quality; or more time of low quality	Continuous flexibility in making communicative temptations
15. Fluency and connectedness	No interaction is established	Interaction lacks smoothness, appears to be largely dominated by one partner	Fluid and balanced interaction that is often sustained
16. Shared routines and rituals	No evidence of routines and rituals	Some shared routines and rituals which are not sustained and do not permeate the interaction	Sustained, varied and nuanced rituals and routines

*Source:* Adapted from Suma, K., Bakeman, R., & Adamson, L.B. (2024). *The Joint Engagement Rating Inventory (JERI)* [Technical Report 25.4]. Georgia State University. Retrieved from https://sites.gsu.edu/bakeman/adamson-memos/

### Data analysis

Data analysis was conducted using IBM SPSS Statistics (version 27) (IBM Corp, 2020). Descriptive statistics were used to summarise caregiver and child demographic variables using median with interquartile range, range or frequency with percentages, as appropriate. As per convention, JERI inter-rater reliability was assessed using weighted Cohen’s kappa, observer estimated accuracy and percent agreement within one scale point. Weighted Kappa values were interpreted as follows: 0–0.20 indicated no agreement, 0.21–0.39 minimal agreement, 0.40–0.59 weak agreement, 0.60–0.79 moderate agreement, 0.80–0.90 strong agreement and above 0.90 as almost perfect agreement (Cohen, 1968; McHugh, 2012; Sim & Wright, 2005). The observer estimated accuracy determined whether weighted Kappa values were adequate, with the goal of 80% or greater. The inter-rater reliability goal for percentage agreement within one scale point was 80%.

The Wilcoxon signed-rank test for paired samples was used to compare change in child behaviours, caregiver behaviours and dyadic engagement across the two time points (pre- versus post-intervention) because we did not assume a normally distributed dataset given the small sample size and heterogeneity. Given the small sample size in this proof-of-principle study, visual summaries of each JERI rating (pre- versus post-intervention) were also generated to show how participant scores changed over time. Line graphs were created that represented the individual participant scores with box plots indicating medians and ranges of scores across the group.

### Ethical considerations

This study was reviewed and approved by both ethical review boards of the University of Cape Town Human Research Ethics Committee (reference number: 301/2015, 468/2019) and Duke University Institutional Review Board (reference number: Pro00103045, Pro00064533).

## Results

### Participants’ characteristics

Full caregiver–child participant characteristics and participants’ flow are outlined in the pilot study by Rieder and colleagues (Rieder et al., 2023), with key relevant details summarised next. Twelve dyads consented to participate in the study and 10 completed the coaching intervention (one withdrew from the study after three coaching sessions, and one was lost to follow-up after all coaching sessions were completed). Median child age at baseline was 53 months. Eleven of the 12 children were male. To inform DSM-5 autism diagnosis, an ADOS-2 Module 1 was completed with 11 children and the ADOS Module 2 with the remaining child. Six of the primary caregivers were mothers, four were fathers and two were grandmothers. Five caregivers completed Grade 12, three obtained a post-Grade 12 diploma and three completed tertiary education. Nine caregivers were married. In terms of financial status, one caregiver reported they were ‘struggling’, six reported they were ‘just getting by’ and five reported they were ‘doing okay’. Ten caregivers reported speaking English to their children at home and two caregivers reported using another language (but did not specify which one).

### Inter-rater reliability

Inter-rater reliability for each JERI item is outlined in Table 2. Weighted Kappa values for 13 of the 16 JERI items ranged from 0.62 to 1, indicating moderate to strong agreement between raters. The observer estimated accuracy of weighted Kappa values, ranged between 83% and 99%. Joint Engagement Rating Inventory items with moderate to strong agreement included *unengaged, object engagement, joint engagement, symbol-infused joint engagement, responsiveness, expressive language level and use, attention to caregiver, following in on child’s focus, affect, communicative temptations* and *fluency and connectedness*. Percentage agreements within one scale point ranged between 75% and 100% across JERI items.

**TABLE 2:** Inter-rater agreement for each Joint Engagement Rating Inventory item.

JERI Items	Weighted Kappa (wtK)	Observer estimated accuracy (%)	% agreement within one scale point
Unengaged	1.00	99	100
Object engagement	0.88	94	94
Joint engagement	1.00	> 99	94
Supported joint engagement	1.00	> 99	94
Coordinated joint engagement	0.00	> 36	94
Symbol-infused joint engagement	0.73	94	94
Responsiveness	0.68	88	88
Expressive language level and use	1.00	> 99	97
Attention to caregiver	0.79	92	91
Scaffolding	0.62	83	81
Following in	0.73	89	88
Caregiver affect	1.00	> 99	100
Language facilitation	0.38	73	75
Communicative temptations	0.75	93	94
Fluency and connectedness	1.00	> 99	100
Shared routines and rituals	0.00	> 99	100

JERI, Joint Engagement Rating Inventory.

### Signals of change

**FIGURE 2:**
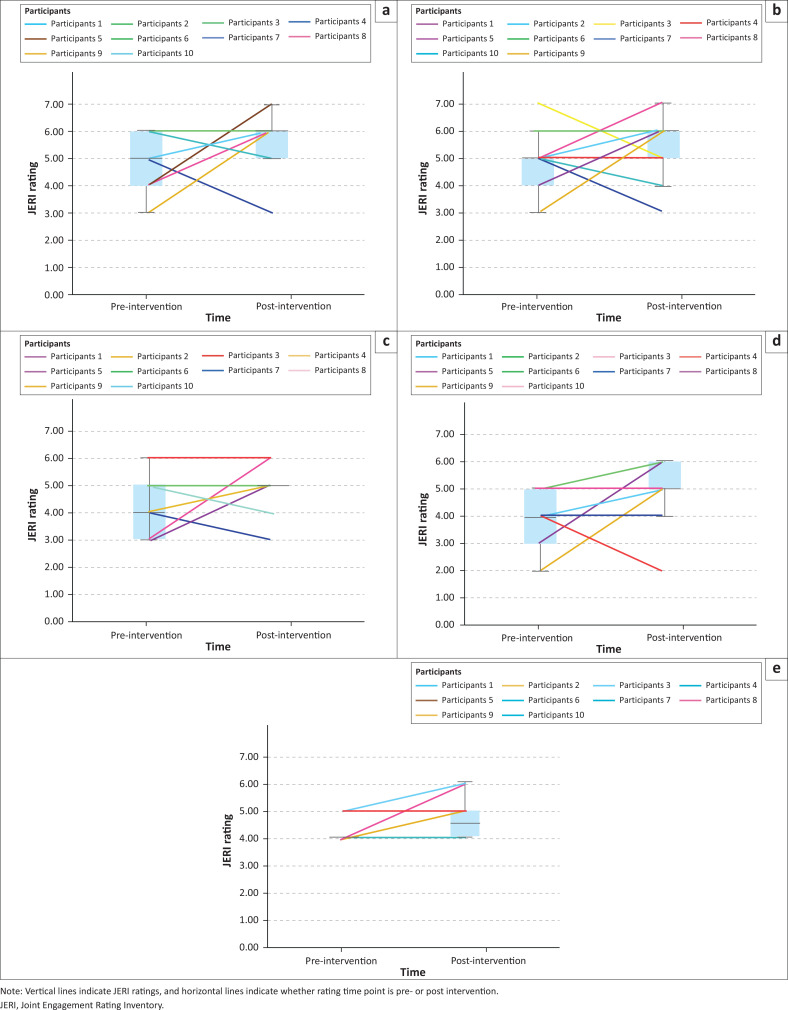
Visual summaries and box plots of caregiver Joint Engagement Rating Inventory items pre- versus post-intervention: (a) Scaffolding, (b) following in on child’s focus, (c) communicative temptations, (d) language facilitation, and (e) caregiver affect.

Joint Engagement Rating Inventory pre–post results for caregiver, child, child activity and dyadic item categories are summarised below. Wilcoxon signed-rank tests are presented in Table 3. Visual summaries of individual participant scores and box plots are shown in Figure 2 to Figure 5.

**FIGURE 3:**
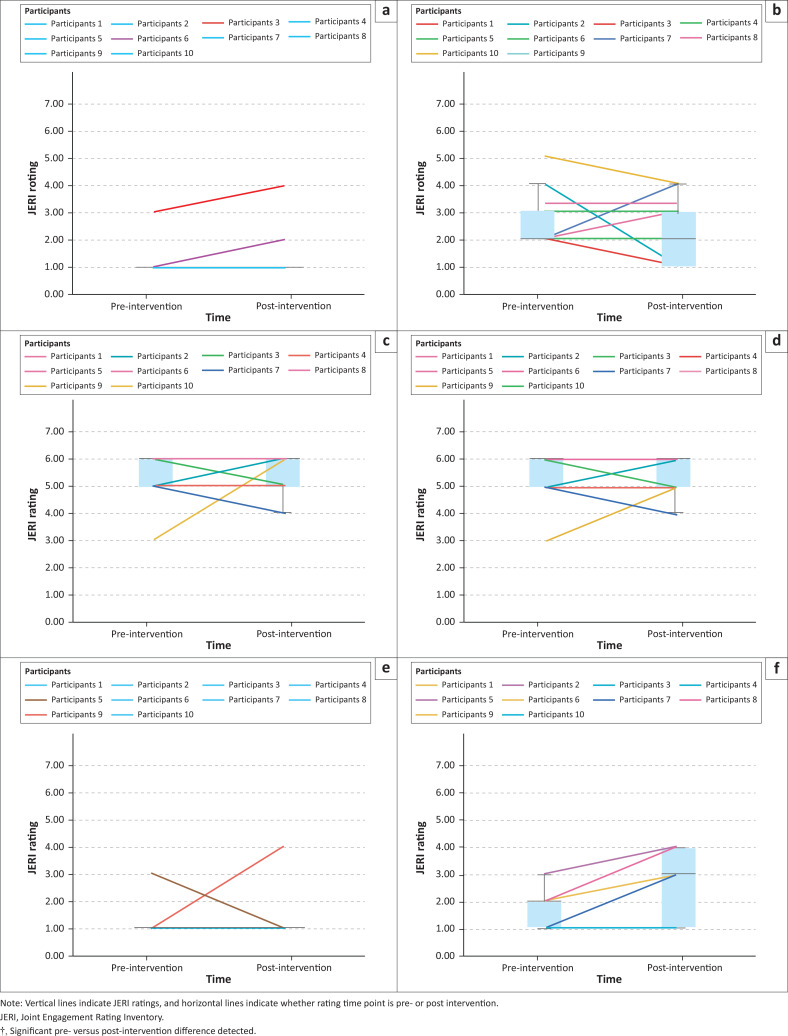
Visual summaries and box plots of child Joint Engagement Rating Inventory items pre- versus post-intervention: (a) Unengaged, (b) object engagement, (c) joint engagement, (d) supported joint engagement, (e) coordinated joint engagement, and (f) symbol-infused joint engagement†.

**FIGURE 4:**
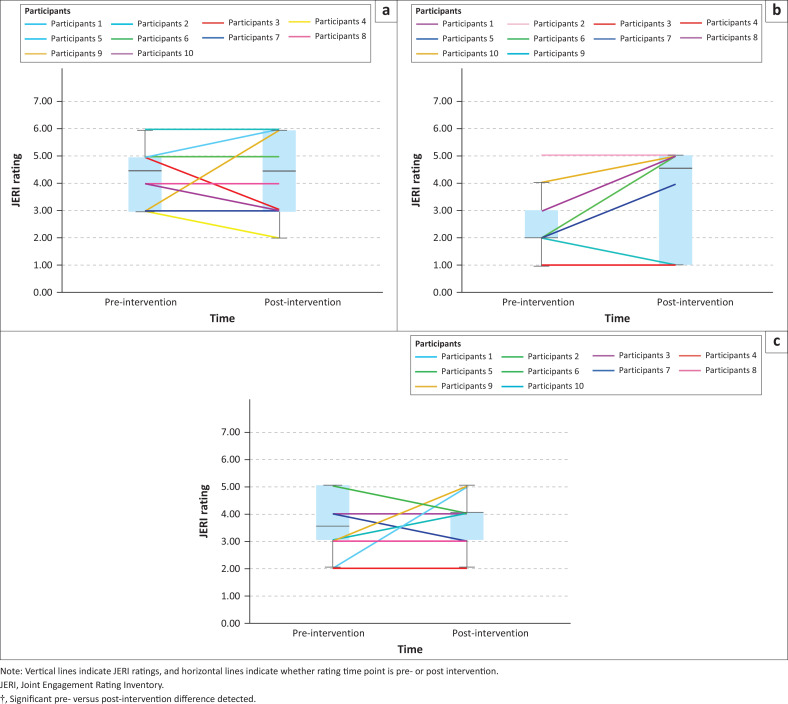
Visual summaries and box plots of child activity Joint Engagement Rating Inventory items pre- versus post-intervention: (a) Responsiveness to partner’s communication, (b) expressive language level and use†, and (c) attention to caregiver.

**FIGURE 5:**
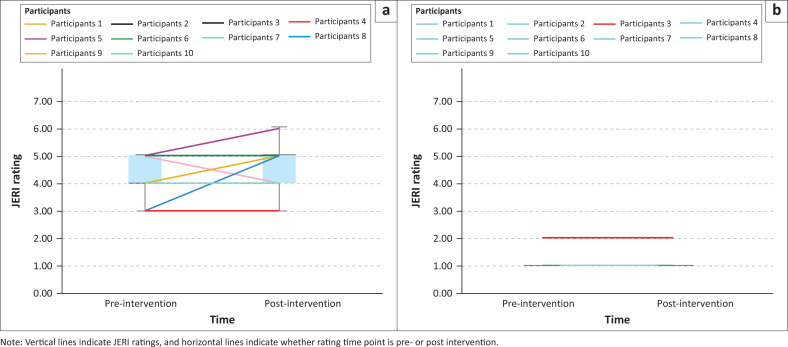
Visual summaries and box plots of dyadic Joint Engagement Rating Inventory items pre- versus post-intervention: (a) Fluency and connectedness, (b) shared routines and rituals.

**TABLE 3:** Wilcoxon signed-rank test results of Joint Engagement Rating Inventory items pre–post intervention.

JERI items	Wilcoxon signed-rank test	
	*z*	*p*
**Caregiver**		
1. Scaffolding	−0.77	0.44
2. Following in on child’s focus	−0.94	0.35
3. Communicative temptations	−1.75	0.08
4. Language facilitation	−1.72	0.09
5. Affect	−1.90	0.06
**Child**		
6. Unengaged	−1.41	0.16
7. Object engagement	−0.65	0.52
8. Joint engagement	0.00	1.00
9. Supported joint engagement	0.00	1.00
10. Coordinated joint engagement	−0.45	0.66
11. Symbol-infused joint engagement	−2.46	0.01
**Child activity**		
12. Responsiveness to partner’s communication	0.11	0.91
13. Expressive language level and use	−2.16	0.03
14. Attention to caregiver	−0.35	0.73
**Dyadic**		
15. Fluency and connectedness	−1.67	0.10
16. Shared routines and rituals	0.00	1.00

JERI, Joint Engagement Rating Inventory.

### Caregiver Joint Engagement Rating Inventory items

Overall, across caregiver JERI items, no significant pre- versus post-intervention differences were detected (see Table 3). However, visual summaries indicate variation in participant-level responses (see Figure 2a–e). In *scaffolding*, four caregivers scored higher, three caregivers scored the same and three caregivers scored lower post-intervention (Figure 2a). For *following in on child’s focus* five caregivers scored higher, two caregivers scored the same and three caregivers scored lower post-intervention (Figure 2b). In *communicative temptations*, six caregivers scored higher, two caregivers scored the same and two caregivers scored lower post-intervention (Figure 2c). For *language facilitation*, six caregivers scored higher, three caregivers scored the same rating at both time points, and one caregiver scored lower post-intervention (Figure 2d). Finally, *caregiver affect* results indicated that six caregivers scored the same ratings at both time points and four caregivers scored higher post-intervention (Figure 2e).

### Child Joint Engagement Rating Inventory items

Across child JERI items, one significant pre- versus post-intervention difference was detected for *symbol-infused joint engagement* (*z* = −2.46, *p* = 0.01) (Table 3). Visual summaries indicate individual variation in responses (Figure 3). For *unengaged*, two children scored higher and the remaining eight children scored the same post-intervention (Figure 3a). In *object engagement*, two children scored higher, four children scored the same and four children scored lower post-intervention (Figure 3b). For *joint engagement* and *supported joint engagement*, two children scored higher, five children scored the same ratings at both time points and three children scored lower post-intervention (Figure 3c and Figure 3d). In *coordinated joint engagement*, one child scored higher, eight children scored the same and one child scored lower post-intervention (Figure 3e). Finally, for *symbol-infused joint engagement*, where a significant change post-intervention was detected, seven children scored higher post-intervention and three children scored the same ratings at both time points (Figure 3f).

### Child activity Joint Engagement Rating Inventory items

Across child activity JERI items, one significant pre- versus post-intervention difference was detected for *expressive language level and use* (*z* = −2.16, *p* = 0.03) (Table 3), with visual summaries indicating individual-level variations across items (Figure 4). For *child responsiveness to partner’s communication*, three children scored higher post-intervention, four children scored the same at both time points and three children scored lower post-intervention (Figure 4a). For *expressive language level and use*, six children scored higher, three children scored the same rating at both time points and one child scored a lower post-intervention (Figure 4b). Finally, for *attention to caregiver*, three children scored higher, three scored the same at both time points and four scored lower post-intervention (Figure 4c).

### Dyadic Joint Engagement Rating Inventory items

Overall, across dyadic JERI items, no significant pre- versus post-intervention differences were detected (Table 3). Visual summaries indicate variation in dyad responses for *fluency and connectedness*, with five caregiver–child dyads scoring higher, four scoring the same at both time points and one scoring lower post-intervention (Figure 5a). For *shared routines and rituals*, visual summaries did not indicate individual score variation, with all dyads scoring the same at both time points (Figure 5b).

## Discussion

The overall goal of this proof-of-principle study was to evaluate whether the JERI could detect signals of change in child behaviours, caregiver behaviours and dyadic engagement in young autistic children and their caregivers, after 12, 1-h NDBI-informed caregiver coaching sessions delivered by non-specialist providers. Two statistically significant differences were detected (for *symbol-infused joint engagement* and *expressive language level and use*). These two group-based significant findings were unexpected given the small sample size in this study. Visual summaries across JERI items showed various change signals in 15 of the 16 ratings pre- versus post-intervention. Taken together, these findings suggest that the JERI has the potential to detect change in the context of a caregiver-mediated intervention. This is encouraging for future early intervention research in South Africa using this behavioural coding approach.

While the goal of this study was to assess whether the JERI could identify signals of change, we can infer from results that some caregivers may have learnt to support and extend their child’s communication abilities and engagement during the CCI. An NDBI caregiver coaching approach can increase caregiver use of key strategies such as scaffolding and following in on a child’s focus of attention, which in turn can impact both dyadic engagement and child behaviours (Mirenda et al., 2022). Furthermore, caregivers’ ability to scaffold their child’s use of materials as well as their communicative bids within extended periods of joint engagement can impact child language and other developmental domains (Wetherby et al., 2014). Of the 10 dyads, six caregivers scored higher for *communication temptations* and *language facilitation* strategies, six children scored higher on *expressive language level and use* and seven children scored higher on *symbol-infused joint engagement* post-intervention. In addition, JERI ratings suggested that some joint activities lasted longer and were of better quality, with dyads more engaged with the materials and each other during the CCI. Of the ten dyads, five scored higher post-intervention for *fluency and connectedness*, which refers to the balance of the joint activity, with both caregiver and child contributing more equally to the interaction. Caregiver use of NDBI strategies can increase the duration and improve the quality of engagement between a caregiver and their child (Beaudoin et al., 2019).

In spite of the small sample size in this proof-of-principle study, two JERI items, namely *symbol-infused joint engagement* and *expressive language level and use* showed a significant change post-intervention. *Symbol-infused joint engagement* requires that a child actively engage in an interaction with others using both receptive and expressive language skills. Therefore, an improvement in this critical engagement state, over time, may lead to growth in child language and cognitive abilities (Adamson et al., 2004). In terms of *child expressive language level*, most children in this study shifted from a limited single-word vocabulary at baseline to two-word utterances over the course of the 12, 1-h caregiver coaching sessions. Even though autism is not a language disorder, delayed expressive language is often the presenting caregiver concern, with approximately 50% of autistic children experiencing delays in this area (Becerra-Culqui et al., 2018). Research shows that child language acquisition is facilitated by foundational skills, including joint attention, shared affect and joint engagement (Schreibman et al., 2015). Thus, coaching caregivers in NDBI strategies that sustain joint engagement with their young autistic child may support growth in child language abilities.

Various JERI items increased post-intervention, but some ratings either stayed the same or decreased post-intervention. There are several possible reasons for this. The strategies caregivers were taught required time to learn and master. Therefore, it may take longer than 12, 1-h sessions to see change in caregiver strategies, child engagement and dyadic interaction (Rogers et al., 2019). Variability in child behaviour may also impact interactions depending on factors such as child affect or mood on the day the CCI was recorded. Along with this, behavioural coding was only conducted on two 6-min CCI videos that were recorded in a lab-based setting, which did not allow for in-depth insight into the dyad’s behaviours, particularly their behaviour within their home environment. This 6-min window may therefore not provide comprehensive representation of child’s and caregiver’s behaviours.

The study has limitations that are important to acknowledge. Firstly, the sample size in this proof-of-principle single-arm study was small, with 10 caregivers and their autistic children completing baseline and follow-up assessments. While the study sample size and design limited power to detect statistically significant group differences, in an effort to increase rigour, raters were blinded to pre- versus post-intervention status of CCI videos coded. Secondly, the CCI were recorded in a lab-based setting. Recording these interactions in a lab setting was considered a reasonable starting point as this study, to our knowledge, was the first of its kind in South Africa to examine the use of the JERI to detect signals of change in response to an NDBI coaching approach. Thirdly, we acknowledge that the cultural sensitivity of the JERI was not examined in this study. However, researchers who coded CCI were from culturally diverse backgrounds, with one rater identifying as a multilingual black South African (speaking Xitsonga, English, isiXhosa, isiZulu, Sesotho and Afrikaans) and the other, a multilingual Indian Malawian living in South Africa (speaking English, Chichewa, Gujarati and French). In addition, a range of low-cost play materials readily available in South African stores were used in the CCI protocol, thus providing dyads with culturally familiar materials.

## Conclusion

There is limited evidence on the effectiveness of NDBI caregiver coaching approaches implemented through task-sharing in culturally and linguistically diverse contexts (Lord et al., 2022). This is an important area of research, firstly, because most autistic people live in diverse contexts and, secondly, because task-sharing can help to address the gap in services and early intervention supports faced by many families around the globe. Study results suggest that it is possible to detect signals of change using the JERI behavioural coding scheme in child behaviours, caregiver behaviours and dyadic engagement after 12, 1-h NDBI-informed caregiver coaching sessions. An important next step would be to include the JERI, as an intervention outcome measure, in larger-scale clinical trials, designed and powered to assess response to intervention. Future research could also examine the impact of sociodemographic factors on a caregiver’s ability to learn NDBI strategies in which they are coached.
